# Diabetes and Stem Cell Function

**DOI:** 10.1155/2015/592915

**Published:** 2015-05-17

**Authors:** Shin Fujimaki, Tamami Wakabayashi, Tohru Takemasa, Makoto Asashima, Tomoko Kuwabara

**Affiliations:** ^1^Research Center for Stem Cell Engineering, National Institute of Advanced Industrial Science and Technology (AIST), Central 4, 1-1-4 Higashi, Tsukuba, Ibaraki 305-8562, Japan; ^2^Physical Education, Health and Sport Sciences, Graduate School of Comprehensive Human Sciences, University of Tsukuba, 1-1-1 Tennodai, Tsukuba, Ibaraki 305-8577, Japan

## Abstract

Diabetes mellitus is one of the most common serious metabolic diseases that results in hyperglycemia due to defects of insulin secretion or insulin action or both. The present review focuses on the alterations to the diabetic neuronal tissues and skeletal muscle, including stem cells in both tissues, and the preventive effects of physical activity on diabetes. Diabetes is associated with various nervous disorders, such as cognitive deficits, depression, and Alzheimer's disease, and that may be caused by neural stem cell dysfunction. Additionally, diabetes induces skeletal muscle atrophy, the impairment of energy metabolism, and muscle weakness. Similar to neural stem cells, the proliferation and differentiation are attenuated in skeletal muscle stem cells, termed satellite cells. However, physical activity is very useful for preventing the diabetic alteration to the neuronal tissues and skeletal muscle. Physical activity improves neurogenic capacity of neural stem cells and the proliferative and differentiative abilities of satellite cells. The present review proposes physical activity as a useful measure for the patients in diabetes to improve the physiological functions and to maintain their quality of life. It further discusses the use of stem cell-based approaches in the context of diabetes treatment.

## 1. Introduction

Diabetes mellitus (DM) is one of the most common serious metabolic diseases that has spread all over the world, and the number of people with diabetes has continued to grow in recent years. The patients with DM represent a hyperglycemic state induced by impairments in insulin secretion (type 1), insulin action (type 2), or both. Type 1 diabetes mellitus (T1DM), which accounts for less than 10% of patients with diabetes, is characterized by an immune-mediated destruction of pancreatic *β* cells in the pancreatic islets of Langerhans, leading to insulin deficiency [1]. It is well known that T1DM is developed in childhood and can lead to severe long-term complications such as retinopathy, neuropathy, and nephropathy, as well as macrovascular diseases, including cerebral, coronary, or peripheral vascular systems [2]. On the other hand, type 2 diabetes mellitus (T2DM), which accounts for over 90% of patients with diabetes, occurs through mechanisms such as insulin resistance in peripheral tissues and increased blood glucose levels induced by overnutrition associated with the deficiency of insulin secretion [3, 4]. DM is often associated with secondary complications that affect multiple organs such as the eyes, kidneys, heart, brain, and skeletal muscle [5].

The central nervous system is notably affected by diabetes. DM has been reported to induce pathological alterations in the nervous system, resulting in the onset of cognitive deficits and an increased risk for vascular complications in the brain [6]. Furthermore, it has been demonstrated that DM is associated with vascular dementia, depression, and Alzheimer's disease (AD) [7–11]. These disorders may be caused by morphological changes, such as white matter leukoaraiosis, as well as hippocampal, cortical, and amygdala atrophies, in the brains of the patients with DM [12, 13]. Additionally, the skeletal muscle is critically influenced by diabetes. It has been reported that DM induces skeletal muscle atrophy [14–16] and fiber-type transition from oxidative to glycolytic [17, 18]. Moreover, the impairment of energy metabolism has been observed in diabetic skeletal muscles [19, 20]. These alterations lead to skeletal muscle dysfunctions, such as muscle weakness and exercise intolerance [16, 21]. Among the multiple factors that can cause the disturbances to the central nervous system and skeletal muscle function, one of the candidates is stem cell dysfunction in DM. Neural stem cells (NSCs) are self-renewing multipotent cells that generate neurons, astrocytes, and oligodendrocytes in the nervous system [22]. It has been reported that the proliferative abilities of NSCs are declined in the hippocampus of T1DM model animals [23, 24]. NeuroD1 is a basic helix-loop-helix transcription factor that promotes neurogenesis [25]. The neurogenesis of NSCs is impaired through the inhibition of the NeuroD1 transcription factor expression in DM [26]. Similar to NSCs, the proliferation and differentiation of skeletal muscle stem cells, termed satellite cells, are attenuated in diabetic muscles [27, 28]. The stem cell dysfunction may induce the impairment of cell turnover, resulting in the disturbed functions of the brain and skeletal muscle in DM.

This review will focus on the alterations to the central nervous system and skeletal muscle in diabetes, including the function of NSCs and satellite cells. Furthermore, we will attempt to clarify the effects of exercise as diabetes prevention and therapy on the brain and skeletal muscle in diabetes.

## 2. The Alteration of Neurogenesis in Diabetes

### 2.1. The Central Nervous System in Diabetes

In both human and animal models, DM is associated with pathological changes in the nervous system that lead to cognitive deficits and to an increased risk for vascular complications in the brain [6]. Patients with diabetes, especially older adults, apparently face a greater risk of vascular dementia. However, according to large population studies, DM is also associated with depression and AD [7–11]. Numerous studies have reported cognitive deficits in both T1DM and T2DM patients, who show slowing of information processing speeds and worsening psychomotor efficiencies and deficits in vocabulary, attention, and memory [5, 29, 30].

Morphological changes have been identified in patients with both T1DM and T2DM diabetes [12, 31–34]. These include global subcortical and cortical atrophies and white matter leukoaraiosis (local white matter intensity changes observed in MRI images). Diabetic patients are more prone to developing extensive and earlier leukoaraiosis [12]. People with more advanced leukoaraiosis are at an increased risk for cognitive impairment and dementia [32, 33]. Also, leukoaraiosis is usually found in the brain scans of elderly people, especially those over 80 years old; therefore, diabetes has been considered to accelerate the aging process. Magnetic resonance imaging (MRI) has also demonstrated that T2DM patients have hippocampal and amygdala atrophies when compared with control subjects [13]. The hippocampus, where the atrophy is found in AD, is responsible for learning and memory functions, suggesting that diabetes is a risk factor for AD.

### 2.2. Adult Neurogenesis

**Figure 1:**
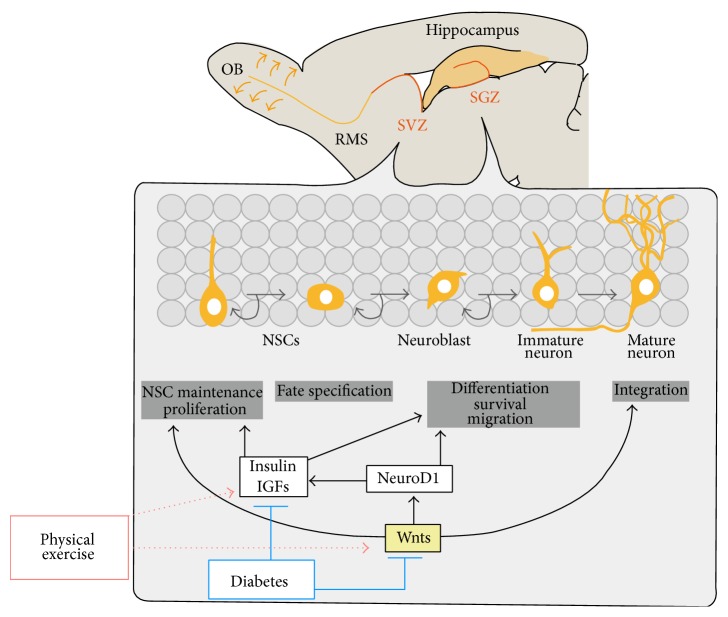
The schematic diagram of regulation of adult neurogenesis by insulin/IGFs and Wnt signals. Adult neural stem cells (NSCs) are primarily located in two distinct regions of the brain: the SVZ of the lateral ventricles and the SGZ of hippocampal dentate gyrus. In the SGZ, adult NSCs undergo proliferation, fate specification, maturation, migration, and eventual integration into the preexisting neural circuitry. In the SVZ, adult NSCs give rise to neuroblasts, which migrate into the olfactory bulb through rostral migratory stream (RMS) and differentiate into mature local interneurons. The progression of NSCs to mature neurons in adult SVZ and SGZ is multistep process with distinct stages and is controlled by insulin/IGFs and Wnts. Diabetes inhibits insulin/IGFs and Wnts signaling in adult neurogenesis, which lead to the decline of adult neurogenesis, while physical exercise may recover diabetes-induced inactivation of Insulin/IGF and Wnt signaling. NSCs, neural stem cells, SVZ, subventricular zone, and SGZ, subgranular zone.

Neurogenesis in the mammalian brain is a multistep process that includes the proliferation of neural progenitor cells, fate determination, migration, neuronal maturation, and the functional integration of newborn cells into the existing neuronal circuitry (Figure 1). Although neurogenesis almost completely ceases after birth, recent studies have demonstrated that it still takes place constitutively at low levels in the adult brain of mammals including rodents, primates, and humans [35–40]. NSCs are present and continuously generate functional neurons specifically in the subventricular zone (SVZ) of the lateral ventricles and the subgranular zone (SGZ) of the dentate gyrus (DG) in the hippocampus. NSCs born in the SVZ become proliferating neuroblasts and migrate through the rostral migratory stream (RMS) into the olfactory bulb. Neuroblasts then become immature neurons and integrate into local interneurons. In the DG of the hippocampus, the proliferating neuroblast cells become immature neurons and project their axons into the CA3 region. The neurons eventually differentiate into mature neurons and are integrated into the preexisting hippocampal circuitry as functional granule cells. Recent studies show that newly formed neurons are incorporated into the functional networks of both the OB and the DG, which suggests significant effects of adult neurogenesis on brain functions associated with learning, memory processing, and odor discrimination [41–45].

In the SVZ and SGZ neurogenic niche, there are astrocytes, endothelial cells, astrocytes, ependymal cells, oligodendrocytes, and mature neurons. A transplantation study indicated the importance of the neurogenic niche in determining the fate of adult NSCs. Neural stem cells derived from the adult hippocampus or spinal cord can give rise to neurons after grafting into the DG, but not into the spinal cord [46]. Moreover, recent work by Song et al. [47] has suggested that specified microenvironments provide the unique neurogenic niche for adult neurogenesis. In this study, astrocytes from the hippocampus provided signals that instructed the adult NSCs to differentiate into neurons, but the astrocytes from the adult spinal cord could not. This evidence suggests that the local environment dictates the fate of adult NSCs, and that astrocyte-derived soluble and membrane-bound factors promote neurogenesis. Other components of the neurogenic niche have been also been identified as supporting neurogenesis, such as endothelial cells [48], microglia [49], and the vascular system [50]. Particularly astrocytes regulate neurogenesis by secreting factors such as Wnt3 and a major proinflammatory cytokine, interleukin-1b. Therefore, the microenvironments of the SVZ and SGZ, but not other brain regions, are thought to possess specific factors that allow the differentiation and integration of new neurons. A variety of molecules serve as niche signals to regulate the maintenance, activation, and fate choice of adult NSCs, including Notch, BMPs, Shh, and Noggin, growth and neurotrophic factors, and Wnts [51]. Through intrinsic and extrinsic factors, adult neurogenesis is tightly regulated in order to allow for the maintenance and self-renewal of the stem cell pool and the generation of fully functional neurons.

### 2.3. The Role of Wnt and Insulin/IGFs Signals in Adult Neurogenesis

Several studies have demonstrated the role of Wnt signaling in adult neurogenesis. For example, Wnt3 is strongly expressed in the astrocytes of the neurogenic niche. NSCs expressed major components of the canonical Wnt/*β*-catenin pathway [52, 53]. Therefore, NSCs could receive Wnt signals and stimulate the canonical Wnt/*β*-catenin pathway. The coculture study of NSCs with the hippocampus astrocytes also showed that astrocyte-derived Wnts stimulated neuroblast proliferation and neuronal differentiation in adult hippocampal NSCs through the Wnt/*β*-catenin pathway [52]. In addition to the astrocyte-derived Wnts, there is an autocrine Wnt signaling in hippocampal NSCs as several Wnts are expressed in the hippocampal NSCs [53]. Interestingly, the inhibition of the autocrine Wnt stimulation promotes neurogenesis and reduces the multipotent progenitors, which indicates that the Wnt autocrine pathway promotes differentiation into neurons, but it also helps in the maintenance of the stem cell pool. The injection of a lentivirus vector expressing Wnt3 or a dominant negative Wnt into the DGs of mice also showed that the activation of Wnt signaling increased the adult neurogenesis, while the inhibition of the Wnt signaling reduced neurogenesis significantly. Furthermore, the inhibition of the Wnt signaling in the DGs of adult rats also demonstrated the impairment of spatial memory and object recognition [54]. These results indicate the profound role of Wnt signaling in adult neurogenesis and its involvement in the cognitive function.

Interestingly, NeuroD1, one of the major targets of Wnts, is selectively expressed in dividing neural progenitors and in immature granule neurons in the adult DG, but not in Sox2-expressing hippocampal NSCs. Furthermore, Kuwabara et al. demonstrated that the NeuroD1 promoter could bind to Sox2 and TCF/LEF, the major downstream transcription factor of the Wnt/*β*-catenin pathway thorough* in silico* analyses of NeuroD1 promoter. According to the study, it is suggested that NeuroD1 transcription is activated by Wnts through TCF/LEF in NSCs, which allow neurogenesis to proceed, while its transcription is silenced by Sox2 that inhibited neurogenesis [25]. In using NeuroD1 conditional knockout (KO) mice, Gao et al. demonstrated that NeuroD1 is required for neurogenesis in the adult hippocampus* in vivo* and* in vitro* [55]. Therefore, Wnt-mediated neurogenesis requires NeuroD1 in adult hippocampal neural progenitor cells. According to these studies, the activation of the canonical Wnt pathway accumulates *β*-catenin, which induces the transcription of NeuroD1 in the NSCs and therefore induces neuronal differentiation. On the other hand, Sox2+ multipotent progenitor cells silence NeuroD1 transcription and maintain undifferentiated NSCs. Our recent study also demonstrated that insulin is synthesized in adult hippocampal neurons, but also in adult NSCs derived from the hippocampus and OB by the induction of neuronal differentiation [56]. According to the study, NeuroD1 is shown to activate the insulin gene expression directly in NSCs from the adult hippocampus and the OB. Thus, under the Wnt3 activation, NeuroD1 promotes neurogenesis but also induces insulin production in adult OB and hippocampal NSCs.

In addition to Wnt3, recent studies provided the evidence that other Wnt factors are involved in the adult neurogenesis. Erickson et al. investigated that Wnt7a has a function to promote the proliferation and self-renewal of adult NSCs through the canonical Wnt signaling pathway in neurogenic regions of the adult brain [57]. Overexpression of Wnt3a and Wnt5a in the adult SVZ was shown to promote the proliferation and neuronal differentiation of adult neural progenitor cells* in vitro* [58]. The stabilization of *β*-catenin by retrovirus-mediated expression was also shown to promote the proliferation of neural progenitor cells in the SVZ* in vivo*, resulting in increased neurogenesis in the OBs [59]. Thus, the regulation of Wnt signaling proves to be essential for adult neurogenesis (Figure 1).

Several studies have reported that insulin/IGF signaling has important role in controlling differentiation of NSCs [56, 60–63]. The activation of insulin/IGF signaling stimulates the proliferation of neural stem cells in the undifferentiated state, induces the differentiation of oligodendrocytes, and increases the survival of neurons and oligodendrocytes [60]. Additionally, IGF-1 signaling is necessary for neuroblast migration from the SVZ, as a result of neuroblast accumulation in the SVZ and improper migration to the OB in IGF-1 KO mice [64]. Therefore, insulin/IGF signaling is necessary for the maintenance of NSCs, cell fate specification, and migration and survival of neurons.

### 2.4. Impairment of Adult Neurogenesis in Diabetes

The hippocampal formation is clearly recognized as being involved in learning and memory. Increasing evidence has shown that diabetes may be associated with deficits in learning and memory. In a pharmacologically induced model of T1DM diabetes, streptozotocin- (STZ-) induced diabetes consistently decreased hippocampal cell proliferation in rodents [23, 24, 65–69]. Through the incorporation of BrdU, immature neurons were demonstrated to decrease significantly in STZ-induced animals [23, 24], which indicates that neuronal differentiation is downregulated in STZ-induced diabetic rats. In addition, the proportion of mature neurons after STZ-induced diabetes in rats was shown to be either decreased [24] or unchanged [67]. In summary, STZ-induced T1DM models consistently decreased hippocampal cell proliferation and survival, and in some investigations the models were also negatively affected by neuronal differentiation. Nonobese diabetic (NOD) mice are another model of T1DM diabetes, which spontaneously develop T1DM through the autoimmune destruction of the pancreatic *β* cells [70]. Similar to the STZ-induced T1DM model, NOD mice showed decreased hippocampal cell proliferation [71, 72] and reductions in neuronal differentiation [72]. Diabetic NOD mice, as well as NOD mice that did not develop diabetes, showed significantly lower levels of cell survival than the controls [71]. Interestingly, neuronal survival was also more increased in the NOD mice that did not become diabetic than in NOD mice that became diabetic at 15 weeks of age, but the rate of cells becoming neurons did not differ between the nondiabetic and diabetic NOD mouse groups. This suggests that the impairment of hippocampal neurogenesis is expected in the NOD mice long before the diabetic features are apparent, and the mice that eventually develop diabetes will exhibit greater reductions in neurogenesis.

Hippocampal neurogenesis has been studied in a number of animal models of T2DM including genetic models in mice and rats, such as the *db*/*db* mouse, the Zucker diabetic fatty (ZDF) rat, the Goto-Kakizaki (GK) rat, and an environmental model of high-fat diet-induced obesity. Both the *db*/*db* mouse and ZDF rat are leptin-receptor deficient and are used as models of obesity complicated by diabetes. The GK rat is a polygenic model with elevated blood glucose, peripheral insulin resistance, a nonobese phenotype, and the exhibition of many degenerative changes observed in human T2DM. The reduction in adult neurogenesis in these models has been demonstrated in many studies [73]. For example, ZDF rats have decreased hippocampal cell proliferation and neuronal differentiation compared to their lean, nondiabetic controls as measured by Ki67 or doublecortin immunoreactivity [74]. Similarly, *db*/*db* mice also demonstrated decreased hippocampal cell proliferation in the diabetic mice compared to the control group [67, 75]. According to these studies, the hippocampal neurogenesis is severely impaired in T2DM.

Diabetic mice with decreased hippocampal proliferation also showed cognitive deficits, as demonstrated by various hippocampus-mediated behavioral tests, including the Morris water maze, novel object recognition, and novel object placement [65, 67, 69]. STZ-induced T1DM models showed deficits involving learning and memory by measuring the Morris water maze and novel object recognition tests [67]. The STZ-induced diabetic rats spent less time identifying the novel object than the control group in novel object recognition, which implies that the STZ-induced diabetic rats incorrectly identified a novel object as familiar. The STZ rats also showed significant deficits in learning the location of the hidden platform in the Morris water maze. Similar deficits have also been observed in *db*/*db* mice [67]. The *db*/*db* mice showed impairments in novelty discrimination when compared to the control, as well as a significantly impaired performance in the Morris water maze. Altogether, the impairment of hippocampal neurogenesis in diabetes accompanied with cognitive deficits in animal models is similar to diabetic patients.

Our previous study provided evidence that the Wnt signaling pathway and NeuroD1 expression are both inhibited in the hippocampus and OBs of STZ-induced diabetic rats [26]. Immature neurons migrate from the SVZ to OBs and become mature neurons. The inhibition of Wnt signaling and NeuroD1 in both neurogenic niches of STZ-induced diabetic animals suggests that the impairment of neurogenesis in diabetes is the result of the inhibition of Wnt signaling (Figure 1). In addition, the inhibition of the Wnt signaling in the DG of adult rats leads to the impairment of spatial memory and object recognition [54]. The inhibition of Wnt signaling may also be attributed to the cognitive deficits in diabetes.

### 2.5. Neurodegenerative Diseases and Diabetes

Neurodegenerative diseases are typically progressive late-onset disorders that lead to impairments in cognition and/or motor function. These diseases share similar features including an abnormal accumulation of protein, including plaques and tangles in AD, Lewy bodies in Parkinson's disease, and nuclear and cytoplasmic accumulations in polyQ diseases like Huntington's disease (HD). Diabetes has been identified as a risk factor for neurodegenerative diseases such as AD and HD.

T2DM has been specifically identified as a risk factor for AD, which is most likely linked to an impairment of insulin signaling in the brain. AD is an age-related neurodegenerative disease associated with the increased production and aggregation of amyloid-*β* (A*β*) peptides and intracellular neurofibrillary tangles of the hyperphosphorylated tau protein in the brain [76]. Recent studies provided the evidence that the insulin receptor and its signaling activities are reduced in the brain with AD [77, 78]. In addition to the impairment of insulin signaling, the brain/neuron-specific insulin-receptor KO mice exhibited a substantial increase in the phosphorylation of the microtubule-associated protein tau, a hallmark of neurodegenerative diseases [79]. In addition, the treatment with antidiabetic agents, including glucagon-like peptide-1 (GLP-1) receptor agonists, exendin-4, and liraglutide, which are approved for the treatment of T2DM, has been shown to facilitate insulin signaling and sequential reductions in the endogenous levels of A*β* in the brain and prevent hippocampal neuronal death [80]. The GLP-1 has been shown to enhance cognitive performance in rodents [81], thus suggesting a protection from neuronal loss and cognitive deficits in AD. Based on these investigations, insulin resistance in AD may contribute to the disease pathophysiology.

HD is an autosomal, dominantly inherited, neurodegenerative disorder characterized by neurological, cognitive, and psychiatric symptoms. Diabetes frequently develops in HD patients [82] and in transgenic mouse models of HD such as the R6/2, HD-N171-82Q mice [83, 84]. The R6/2 mice exhibited cognitive impairments [85] and deficits in the replication of *β* cells and insulin secretion [86], which indicates that HD is expressed not only in neurons but also in the pancreatic islets. Recent studies have demonstrated that hippocampal NeuroD1 expressions were impaired in R6/2 mice [87] and a significant reduction in hippocampal cell proliferation has been observed in R6/2 mice and another HD model mice, R6/1 [88], suggesting the impaired neurogenesis-induced cognitive deficits.

Interestingly, NeuroD1 is known to be expressed in pancreatic *β* cells and to play an essential role in endocrine pancreatic development, as NeuroD1 KO mice exhibited severe diabetes and died perinatally [89]. Furthermore, our previous study provided the evidence that Wnt3 is expressed in pancreatic *α* and its expression is decreased in STZ-induced diabetic rats [56]. Because Wnt3-induced NeuroD1 is a critical factor for neurogenesis and pancreatic development, it is suggested that the cognitive deficit and impairment of insulin secretion in diabetes, AD, and HD are due to the impairment of Wnt3-induced NeruoD1 activity. In summary, recent studies suggest that insulin accelerates AD-related pathologies through its effects on the A*β* metabolism and tau phosphorylation. In addition, HD exhibits the similar diabetic features of insulin secretion deficiency and cognitive deficits. Insulin regulates peripheral energy homeostasis but it also helps the proliferation and differentiation of neuronal precursor cells in the brain. As there are many functions of insulin, the impairment of insulin function leads to multiple deficits in both the brain and the peripheral tissues that could be associated with other neurodegenerative diseases.

## 3. The Alteration to the Satellite Cell Function by Diabetes

### 3.1. The Response and Adaptation of the Skeletal Muscle to Diabetes

The skeletal muscle is the most important organ for insulin action; therefore, the impairment of insulin action can induce various changes in the former including structural, functional changes. The main structural change in skeletal muscles induced by diabetes is muscle atrophy. It is well known that the diabetic muscles lead to the loss of muscle mass. For example, the myofiber diameters of the soleus and extensor digitorum longus in STZ-induced diabetic rats, which is the model of T1DM, decreased by about 30% compared to the control rats [90]. Further studies demonstrated that diabetes induces skeletal muscle atrophy [14–16]. Furthermore, there is increased protein degradation along with decreased protein synthesis in the skeletal muscles in STZ-induced diabetic rats [91], which may be responsible for the loss of muscle mass in diabetes, and muscle atrophy is observed in the T2DM patients. Huang et al. evaluated the skeletal muscle masses of T2DM patients using MRI and showed the reductions in the muscle masses of DM patients [92]. Pedersen et al. also demonstrated that the weight of the appendicular skeletal muscle is significantly lower in DM patients than in the controls [93]. These results suggest that T2DM leads to muscle atrophy, as with T1DM.

Additionally, diabetes induces a muscle fiber-type transition in the skeletal muscle. The skeletal muscle is composed of muscle fibers, which are roughly classified into three types: type I, type IIa, and type IIb [94]. A type I fiber is a slow-twitched oxidative fiber, a type IIb fiber is a fast-twitched glycolytic fiber, and a type IIa fiber is an intermediate fast-twitched oxidative glycolytic fiber [94]. In general, the muscle fiber type shifts from a fast fiber to a slow fiber with increased activity of the skeletal muscle, such as electrostimulation and exercise, whereas inactivity of the skeletal muscle, such as casting and denervation, leads to the transition from a slow fiber to a fast fiber [94]. In the diabetic condition, the shift between muscle fiber types from slow to fast is due to an inactive condition. Hickey et al. demonstrated that DM patients have a significantly lower percentage of type I muscle fibers compared with the control subjects [17]. Oberbach et al. also showed a 16% reduction in slow-fiber fraction and a 49% increase in fast-fiber fraction in diabetic patients [18]. These results may indicate that the oxidative capacity is reduced in diabetic skeletal muscles. In addition to the fiber-type transition, diabetes induced a variety of alterations to the physiological systems in the skeletal muscle, such as vascular changes [95–98], neuropathy [99, 100], and ultrastructural changes [101].

These alterations to the skeletal muscle's structure by diabetes are often associated with reductions in muscle function. Previous studies have demonstrated that diabetes induces muscle weakness and a decline of exercise performance. Regensteiner et al. evaluated exercise performance using a graded treadmill protocol, concluding that the exercise times of the DM patients were significantly lower than those of the sedentary subjects [21]. Kamei et al. demonstrated that FOXO1 transgenic mice, which have insulin resistance, also have a lower exercise tolerance compared to the controls [16]. The diabetes-induced muscle weakness was caused not only by structural changes but also by functional changes in the skeletal muscle. Insulin-stimulated glucose uptake is important for muscle contraction. Thus, diabetes-induced impairment of insulin functions inhibits glucose uptake into the skeletal muscle, resulting in the disturbances to muscle contractions. Cameron et al. reported that diabetic muscles have 15–29% reductions in sciatic motor and sensory saphenous nerve conduction velocities [102]. In addition, DM also leads to a decline in muscle strength. Andersen et al. demonstrated that diabetic patients had 17% and 14% reductions in ankle flexor and ankle extensor strengths, respectively [103]. Sayer et al. also reported that the grip strengths of diabetic patients were significantly lower than those of the control subjects [104]. Moreover, DM induced reductions in endurance capacities. A reduced time to exercise exhaustion was shown in T1DM or T2DM patients compared with nondiabetic subjects [105, 106]. Altogether, diabetes-induced alterations to the skeletal muscle structure and function can lead to muscle weakness and a decline in exercise performance.

The skeletal muscle is the biggest organ to produce energy for biological activity. It is also well known that diabetes alters the energy metabolism in the skeletal muscle. It was observed that diet-induced thermogenesis, which is expressed as a percentage of energy intake, is reduced in DM patients [19], and the maximal oxygen consumption is reduced [20]. The reduced capacity of oxygen consumption may be one of the early phenomena of diabetes-induced energy metabolism disturbances [107]. The impairment of the energy metabolism in DM patient can be caused by multiple factors, and one of the candidates is the functional capacity of mitochondria, which produces adenosine triphosphate (ATP) through respiration and regulates cell metabolism in the skeletal muscle. The mitochondria protein synthesis rate and mitochondria enzyme activities are reduced in DM patients [108]. Furthermore, it was reported that the storage of glycogen in the diabetic skeletal muscle was significantly reduced compared with the controls [109], which is caused by the impairment of glycogen synthase activity. There is a report that glycogen synthase I activity and the content of glucose 6-phosphate, which is an intermediate metabolite produced by hexokinase in the first step of glucose uptake, were significantly increased after insulin injection into the skeletal muscles of nondiabetic rats, whereas a reduced reaction was observed in those of diabetic rats [110]. These results suggest that diabetes induces a disturbance to the glucose metabolism in the skeletal muscle. In addition to glucose metabolism, diabetes leads to impaired lipid oxidation and protein degradation (negative protein balance) [111, 112], resulting in the impairment of energy metabolism.

### 3.2. Skeletal Muscle Stem Cells: Satellite Cells

The skeletal muscle is an abundant source of adult stem cells. Skeletal muscle-specific stem cells, termed satellite cells, contribute to the postnatal maintenance, growth, repair, and regeneration of the skeletal muscle [113]. Satellite cells are characterized anatomically by their location between basal lamina and plasma membranes of muscle fibers and functionally by their myogenic differentiation [114]. In adult skeletal muscles, satellite cells are in a quiescent state under normal physiological conditions. However, in response to muscle injury or exercise, satellite cells are activated and then can proliferate, undergo self-renewal, and differentiate into mature new fibers [115]. A previous study investigated the contribution of satellite cells to skeletal muscle regeneration using satellite cell-depleted mice, and then the satellite cell-depleted skeletal muscle could not be repaired after injury [116]. Thus, satellite cells are essential for the regeneration after a muscle injury.

**Figure 2:**
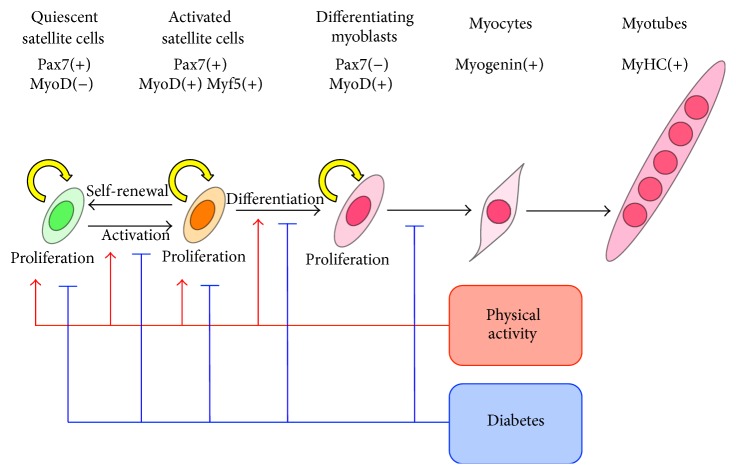
The schematic diagram of regulation of satellite cell activation and differentiation into myotubes. Adult skeletal muscle stem cells (satellite cells) are located between the basal lamina and the myofiber plasma membrane. Although satellite cells are mainly in a quiescent state, they are activated in response to muscle injury or exercise. Activated satellite cells can proliferate, undergo self-renewal, and differentiate into myoblasts and then to myocytes. Myocytes can mutually fuse and generate myotubes. The phases of satellite cell are determined by the expression of marker genes. Quiescent satellite cells express Pax7 (a stem cell-specific transcription factor) alone, whereas activated satellite cells coexpress Pax7, Myf5, and MyoD, which are key transcription factors for myogenic differentiation. Diabetes impairs satellite cell proliferation and activation, resulting in the inhibition of terminal differentiation. However, physical activity (exercise) induces satellite cell activation and improves its proliferative ability. Therefore, physical activity may recover the impairment of satellite cell function in diabetic skeletal muscle.

Satellite cells demonstrate two states in a skeletal muscle turnover: a quiescent state and an activated state (Figure 2). Both quiescent and activated satellite cells express a characteristic marker, Pax7 [117], whereas only activated satellite cells also express Myf5 and MyoD, which are key transcription factors of myogenic lineage progression and differentiation [117]. Although most activated satellite cells proliferate and differentiate through downregulation of Pax7, others withdraw from the cell cycle and return to a quiescent state [118]. The transcription factor Pax7 upregulates the Myf5 expression through the recruitment of the histone methyltransferase (HMT) complex, and then the complex directly methylates histone H3 lysine 4 (H3K4) on the promoter of the Myf5 locus [119]. Pax7(+)/Myf5(+) coexpressing satellite cells upregulates the MyoD expression [120], and then MyoD initiates the transcription of myogenin and other muscle-specific genes [121]. MyoD may be a master regulator of myogenesis to induce the upregulation of muscle-specific gene transcription.

### 3.3. Impairment of Satellite Cell Function in Diabetes

Previous studies have shown that diabetes induces the impairment of satellite cell function. First, T1DM leads to an impairment of the regenerative capacity of the skeletal muscle caused by a decline in satellite cell function. Jeong et al. reported that satellite cells derived from STZ-induced diabetic mice fail in their abilities to form myotubes, resulting in an impairment in regeneration following a cardiotoxin-induced muscle injury [122]. Aragno et al. showed that the MyoD and myogenin expressions are reduced in the gastrocnemius muscles of STZ-induced diabetic rats [27]. Furthermore, diabetic Akita mice demonstrated attenuated muscle regenerations following injury through an impairment of macrophage infiltration and satellite cell recruitment into degenerative fibers [123]. Although the investigations into satellite cells in T1DM remain poor, some evidences indicate that insulin action is important for maintaining satellite cell function. A better understanding of the alterations to the satellite cell population and function in T1DM are needed for the development of clinical therapeutics in muscle health.

Second, although the reports investigating satellite cell function in T2DM are limited, there are some investigations into the alterations to satellite cell function in the conditions of hyperglycemia and lipotoxicity. Hu et al. reported that insufficient muscle regeneration was observed after cardiotoxin injuries in mice fed high-fat diets for 8 months [124]. Woo et al. also demonstrated that 3 weeks of high-fat diet feeding induced a reduction in the number of satellite cells, as well as the impairment of muscle regeneration [125]. Furthermore, an* in vitro* study reported that satellite cells cultivated in a growth medium with a high glucose content tended to differentiate into adipocytes [126], suggesting that the myogenic capacities of satellite cells may be influenced by diabetes. Additionally, ZDF rats (typically used as model of metabolic syndrome) showed the reductions in satellite cell proliferations with no changed proportions of quiescent satellite cells [127]. This study also indicated that MyoD and myogenin protein levels decreased in the plantaris muscles of ZDF rats compared with the control lean Zucker rats [127]. Similarly, transgenic *ob*/*ob* and *db*/*db* mice, which are common mouse models of T2DM, displayed impaired satellite cell proliferation and muscle regeneration [28]. In addition to the myogenic potential, satellite cells derived from DM retain diabetic phenotypes, such as increased expressions of inflammatory cytokines [128], reduced lipid oxidation [129], disturbed glucose uptake [130], and insulin resistance [131]. These results suggest that T2DM and obesity promote various impairments of satellite cell function such as proliferation and differentiation.

Although it is clear that diabetes induces the impairment of satellite cell function, understandings of the molecular mechanisms remain insufficient. One of the candidates of the satellite cell dysfunction in DM is oxidative stress. In both T1DM and T2DM, oxidative stress in the skeletal muscle is elevated in association with glucose concentration [27, 132, 133]. Evans et al. reported that a redox imbalance is induced in the pathogenesis of diabetes and its complications [134]. Additionally, an* in vitro* study demonstrated that a culture of human satellite cells with ROS-inducing hydrogen peroxide (H_2_O_2_) reduced cell lifespans and proliferative capacities [135]. These results suggest that oxidative stress contributes to the impairment of myogenesis in diabetes. In addition to the oxidative stress, there are inflammatory factors as other candidates of the satellite cell dysfunction in DM. In T1DM and T2DM patients, the level of circulating interleukin-6 (IL-6), which is a proinflammatory cytokine playing an important role in skeletal muscle metabolism through its receptor, is increased [136, 137]. A transient elevation of IL-6 leads to satellite cell proliferation, whereas a chronic elevation of IL-6 induces the impairment of satellite cell function [137]. Therefore, chronically elevated IL-6 in DM may be responsible for diabetic satellite cell dysfunction. In this manner, there are various factors of diabetic satellite cell dysfunction, whereas further investigation is needed for a better understanding.

## 4. Improved Function of Stem Cells by Exercise

### 4.1. Recovery of Adult Neurogenesis by Exercise

A number of studies have shown that physical exercise enhances the adult neurogenesis [138–144]. According to Van Praag et al., the voluntary physical exercises of young adult (3-month-old) mice promoted cell proliferation, cell survival, and neurogenesis within the DG [138]. Other studies also demonstrated the exercise-mediated increases in neurogenesis in the DGs of the hippocampus in young, adult, and aged animals [139–144]. Moreover, running has been shown to improve the cognitive functions in aged mice and humans [145–147]. Thus, it is suggested that exercise enhances adult neurogenesis, which may contribute to cognitive functions.

Although few studies have examined the effectiveness of exercise in adult neurogenesis in diabetes, these studies demonstrated that exercise has a positive effect on adult neurogenesis in STZ rats [148, 149]. Physical exercise had no particular effects on the blood glucose levels and body weights of diabetic rats. The reduction of hippocampal cell proliferation observed in STZ-induced diabetic rats has increased significantly through treadmill exercise as well [148]. In addition, physical exercise recovered the cognitive deficits in STZ-induced diabetes by measuring the novel object recognition task [149]. These studies provide evidence that physical exercise improves neurogenesis and cognitive deficits in diabetes, which suggests that physical exercise helps the recovery of diabetic complications in the central nervous system.

Exercise has recently been shown to modulate the expressions of genes involved in the Wnt signaling [150]. Moreover, running was found to increase the expression of Wnt3 in the astrocytes of the DGs significantly and to increase the population of Wnt3-expressing cells in young and aged mice [151]. Altogether, it is suggested that physical exercise in diabetes may promote neurogenesis through the activation of Wnt3 and Wnt signaling even though the role of Wnt3 in exercise-induced increases in neurogenesis in diabetes has not been studied yet (Figure 1).

### 4.2. The Response of the Diabetic Muscle to Exercise

The skeletal muscle has a high plasticity and it is affected by various external stimuli. Among a variety of external stimuli, exercise is the best measure to prevent muscle atrophy, because it brings about large effects and it can be performed relatively easily by many people. Many studies demonstrated that exercise causes skeletal muscle hypertrophy [152, 153], and the effects of exercise on the prevention of muscle atrophy have been reported [154, 155]. Fluckey et al. reported that resistance exercises inhibit the loss of skeletal muscle masses in rodents subjected to tail suspension, which is a model of muscle atrophy in which rodents have no grounding stimulation to their hindlimbs [154]. Fujino et al. also showed that low-intensity treadmill running has a protective effect on tail-suspension-induced muscle atrophy [155].

In addition to the preventive effects of muscle atrophy, exercise has some effects on diabetes prevention and therapy. It is well known that exercise increases the insulin sensitivity of the skeletal muscle in patients with T2DM [156]. Furthermore, Meex et al. reported the improvements in insulin sensitivity following exercise accompanied by improved mitochondrial function [157]. Consistent with these results, a number of studies demonstrated that endurance training improves mitochondrial function, maximal oxygen uptake, and insulin sensitivity in patients with T2DM [158–160]. However, it has been reported that the response to exercise is attenuated in the insulin-resistant patients. de Filippis et al. have shown that the exercise-induced upregulation of PGC-1*α*, which is a major regulator for mitochondria biogenesis, is significantly lower in obese subjects than in control subjects [161]. Similarly, some studies have reported the lack of training effects in patients with T2DM [162, 163]. Additionally, high-intensity training has similar effects on endurance training in the improvement of maximal oxygen uptake and mitochondrial function in a healthy skeletal muscle [164, 165]. In the patients with T2DM, two weeks of high-intensity training increased mitochondrial function and blood glucose profiles [166]. From the above, exercise may be a very useful measure to prevent alterations to skeletal muscle mass and functions in DM.

### 4.3. Recovery of Satellite Cells Function by Exercise

Although there are few studies about the effects of exercise on the satellite cells in diabetic skeletal muscles, it is well known that exercise has positive effects on satellite cells. Several studies demonstrated that the number of satellite cells increases after acute or chronic exercises [167, 168]. This increase in satellite cell numbers is also observed in human skeletal muscle [169]. The long-term effects of exercise on satellite cells are apparent in the skeletal muscle of well-trained power lifters, who have 70% more satellite cells than the control subjects [170]. The increased number of satellite cells after training gradually decreases during detraining [169], suggesting that a continuation of exercise is required for maintaining an abundant pool of satellite cells in the skeletal muscle. There is a report indicating that the intensity, rather than duration, of exercise is important for the accretion of the satellite cell pool [171]. As for the exercise style, the most effective method for increasing or maintaining the pool of satellite cells is still being investigated [172]. These results suggest that exercise may contribute to recovering the reduction of satellite cell numbers in DM.

It is reported that exercise is useful not only to the increase in satellite cell numbers but also to the satellite cell activation. Fujimaki et al. demonstrated that 4 weeks of voluntary wheel running induces the upregulation of Wnt signaling, which contributes to facilitating myogenesis and the activation of satellite cells in the skeletal muscles of mice [173]. Consistent with this study, Aschenbach et al. reported that acute treadmill running induces the upregulation of *β*-catenin, which is a key transcription coactivator in Wnt signaling, and this is done through the downregulation of GSK-3*β* [174]. Armstrong and Esser suggested that functional overload, which is a model of resistance training and induced muscle hypertrophy, has revealed activated *β*-catenin in the plantaris muscle [175]. Using immunoprecipitation assay, Fujimaki et al. also showed that the exercise-induced upregulation of Wnt signaling directly modulates the chromatin structures of both the* Myf5* and* MyoD* genes and accelerates their transcription in adult satellite cells, resulting in increases in the mRNA expressions of* Myf5* and* MyoD* and the activation of satellite cells [173]. These results suggest that exercise may inhibit the disturbance of satellite cell function by DM, such as proliferation and differentiation, whereas the effects of exercise on satellite cells in diabetic muscles remain unclear.

## 5. Stem Cell Therapy for Diabetes

Embryonic stem cells are potential sources for insulin producing *β* cell replacement and these transplantations have been proposed as a potential treatment for diabetes. A previous study by D'Amour et al. showed preliminary success at *β* cell differentiation from human embryonic stem cells (hESCs) [176]. However, these cells were not functionally matured so that they coexpressed glucagon and insulin and failed to increase insulin secretion in response to high ambient glucose levels. Interestingly, implantation of these immature cells into mice promoted the generation of mature *β* cells and prevented STZ-induced hyperglycemia [177]. In addition, recent findings demonstrated that the generation* in vitro* of more mature *β* cells from stem cells is possible before transplantation. Using a complex multistep method, hESCs could generate *β* cells that are very similar to the cells in the human pancreas [178, 179]. Contrary to *β* cells from earlier studies, these differentiated *β* cells express insulin, but not other pancreatic hormones, and contain mature insulin granules. Although the latter were not functionally the same as the cells in the human pancreas, they were able to respond to repeated glucose stimulation with increased insulin secretion. Furthermore, transplantation of these cells into T1DM mice maintained normal blood glucose levels for several weeks. Recent progress in hESC research suggests the use of these cells as a potential approach for diabetic treatment in the future, but further studies are still required for their clinical use.

Additionally, it was shown that the transplantation of various stem cells into skeletal muscle may be useful for alleviating diabetic symptoms. Himeno et al. reported that transplantation of mesenchymal stem cell- (MSC-) like cells derived from mouse induced pluripotent stem cells (iPSCs) into hindlimb muscles inhibits the decrease in the capillary number in the transplanted hindlimb muscles of STZ-induced diabetic mice [180]. Okawa et al. showed that transplantation of neural crest-like cells derived from iPSCs into hindlimb muscles ameliorates the reduction of nerve conduction velocity, intraepidermal nerve fiber density, sensitivity to thermal stimuli, sciatic nerve blood flow, plantar skin blood flow, and capillary number-to-muscle fiber ratio by STZ-induced diabetes [181]. This study also reported that the engrafted cells produce growth factors: nerve growth factor, vascular endothelial growth factor, and basic fibroblast growth factor. Furthermore, Naruse et al. demonstrated that the intramuscular injection of endothelial progenitor cells derived from cord blood mononuclear cells into hindlimb skeletal muscles significantly inhibited the impairment of nerve and vascular function in the transplanted limb muscle of diabetic mice [182]. This group also reported that bone marrow-derived MSCs improved the diabetic polyneuropathy in skeletal muscle [183]. In addition, several studies showed that transplantation of human skeletal myoblasts (hSkMs) into hindlimb muscles improves insulin sensitivity and attenuates hyperglycaemia in KK mice, which is a model of T2DM [184, 185]. In conclusion, stem cell transplantation has the potential to become a useful method for treatment of DM, although more investigation is still needed.

## 6. Conclusion

The present review described the diabetes-related changes in various tissues as well as adult stem cell functions. In particular, the central nervous system and skeletal muscle are most affected by DM. In neuronal tissue, neurogenesis is attenuated in diabetes by the downregulation of Wnt signaling, and diabetes is associated with a number of neurodegenerative diseases. In the skeletal muscle, diabetes leads to a variety of structural, functional, and metabolic changes, such as muscle atrophy, muscle weakness, and the reduction in energy turnover. Satellite cell dysfunction is also induced by diabetes, resulting in a disturbance to myogenesis. Exercise is very useful for preventing in diabetes-related alterations to the neuronal tissue and skeletal muscle. The effects of exercise implicate the upregulation of Wnt signaling, resulting in the activation of neurogenesis in adult neuronal tissue and myogenesis in mature skeletal muscles. Although more investigation is required for a thorough understanding of diabetes-related changes and their biological mechanisms in a variety of tissues, this review proposes exercise as a useful measure for DM patients to prevent the negative effects of diabetes and to maintain their quality of life.
